# Failure to Upregulate the RNA Binding Protein ZBP After Injury Leads to Impaired Regeneration in a Rodent Model of Diabetic Peripheral Neuropathy

**DOI:** 10.3389/fnmol.2021.728163

**Published:** 2021-12-07

**Authors:** James I. Jones, Christopher J. Costa, Caitlin Cooney, David C. Goldberg, Matthew Ponticiello, Melanie W. Cohen, Wilfredo Mellado, Thong C. Ma, Dianna E. Willis

**Affiliations:** ^1^Burke Neurological Institute, White Plains, NY, United States; ^2^Feil Family Brain & Mind Research Institute, Weill Cornell Medicine, New York, NY, United States

**Keywords:** axon, regeneration, RNA-binding protein, ZBP, neuropathy, DPN

## Abstract

Most diabetes patients eventually suffer from peripheral nerve degeneration. Unfortunately, there is no treatment for the condition and its mechanisms are not well understood. There is, however, an emerging consensus that the inability of peripheral nerves to regenerate normally after injury contributes to the pathophysiology. We have previously shown that regeneration of peripheral axons requires local axonal translation of a pool of axonal mRNAs and that the levels and members of this axonal mRNA pool are altered in response to injury. Here, we show that following sciatic nerve injury in a streptozotocin rodent model of type I diabetes, this mobilization of RNAs into the injured axons is attenuated and correlates with decreased axonal regeneration. This failure of axonal RNA localization results from decreased levels of the RNA binding protein ZBP1. Over-expression of ZBP1 rescues the *in vitro* growth defect in injured dorsal root ganglion neurons from diabetic rodents. These results provide evidence that decreased neuronal responsiveness to injury in diabetes is due to a decreased ability to alter the pool of axonal mRNAs available for local translation, and may open new therapeutic opportunities for diabetic peripheral neuropathy.

## Introduction

Axonal injury in the peripheral nervous system (PNS) conditions neurons for more rapid axonal regeneration; these neurons show more rapid elongation after a subsequent injury (McQuarrie, 1978; Forman et al., 1980; McQuarrie and Grafstein, 1981; Lankford et al., 1998). Nerve injury is accompanied by a characteristic increase in the levels of many extracellular factors, such as neurotrophins and cAMP, which are known to enhance axonal growth. Neurons are large, polarized cells with morphologically and functionally distinct sub-cellular domains that are determined by the proteins that reside in these regions (Craig and Banker, 1994). mRNA sorting and localized protein synthesis contribute to the targeting of proteins to specific sub-cellular domains of neurons, including the axonal compartment, which has the ability to locally generate new proteins. Indeed, studies from several groups, including our own, indicate that the capacity for intra-axonal protein synthesis is retained into adulthood (Koenig et al., 2000; Zheng et al., 2001; Hanz et al., 2003; Perlson et al., 2005; Verma et al., 2005; Willis et al., 2005, 2007; Gumy et al., 2011). Our results show that several hundred mRNAs extend into adult rodent dorsal root ganglion (DRG) axons and that the breadth of axonally synthesized proteins is much more complex than initially predicted (Willis et al., 2005, 2007; Gumy et al., 2011). We have also shown that regenerating axons of injury-conditioned (DRG) neurons are capable of local protein synthesis during rapid axonal regeneration (Willis et al., 2005, 2007). Together, the results of these studies indicate that localized protein synthesis plays a critical role in axonal regeneration.

Axonal protein synthesis can be regulated by extracellular stimuli such as chemotropic signaling (Campbell and Holt, 2001, 2003; Brittis et al., 2002; Ming et al., 2002; Brunet et al., 2005; Piper et al., 2005; Wu et al., 2005). Some mRNAs are present in mature axons prior to any stimulation from the environment, and other mRNAs are upregulated only after specific extracellular cues. Among the triggers for axonal mRNA localization is injury. Following sciatic nerve injury, the mRNA encoding β-actin is increased in the axon. As is common for the sub-cellular localization of many mRNAs, the information ‘tagging’ an mRNA for axonal localization is inherent to its sequence (Bassell and Kelic, 2004). For β-actin, binding of the RNA-binding protein (RBP) ZBP1 to the “zipcode” element in its 3’UTR assembles this transcript into granules and directs its axonal localization (Zhang et al., 2001; Gu et al., 2002; Tiruchinapalli et al., 2003). The 3’UTR of rat β-actin mRNA, with its zipcode, is sufficient for regulated axonal transport in response to both attractive and repulsive axonal guidance cues (Willis et al., 2007) and the increased axonal levels of β-actin mRNA following injury is dependent on *cis*-elements and *trans*-acting factors interaction. Reduction in the availability of ZBP1 results in a decrease in the axonal levels of its cargo mRNAs, including β-actin (Donnelly et al., 2011). Regulation of the levels of ZBP1, then, is critical to the successful regeneration of axons following injury.

The induction of regeneration associated gene (RAG) expression by axon injury indicates an active process by which injured neurons sense axonal damage to activate an adaptive response. This can be achieved by the disruption of the retrograde flow of target-derived trophic signals (i.e., loss of NGF; Raivich et al., 1991; Gold et al., 1993), activation of existing or newly synthesized local (axonal) factors that are retrogradely transported (*i.e*., DLK, JNK, STAT3, CREB; Hanz et al., 2003; Cavalli et al., 2005; Cox et al., 2008; Ben-Yaakov et al., 2012; Shin et al., 2012), and depolarization of the axon due to the disruption of the plasma membrane (i.e., Ca^2+^ influx leading to cAMP elevation; Ghosh-Roy et al., 2010; Cho et al., 2013). Ultimately, the arrival of these signals to the cell body drives the transcriptional changes that initiate a regenerative response. Much of the focus of this transcriptional response has been on RAGs that are known to enhance the intrinsic growth capacity of axons; however, given the functionally coordinated nature of this response, it seems likely that other upregulated genes, such as RBPs, are critical to the regeneration response. Failure of this response would be predicted to decrease axonal regeneration.

At least two-thirds of diabetic patients show symptoms of peripheral nerve damage, making peripheral neuropathy one of the most frequent and debilitating complications of diabetes mellitus. Whereas the mechanisms underlying diabetic peripheral neuropathy remain largely unknown, nerve biopsies from diabetic patients show evidence of axonal degeneration and incomplete regeneration, demyelination, and microangiopathy (Dyck and Giannini, 1996). Despite the high incidence, morbidity and mortality, and economic burden of diabetic peripheral neuropathy (DPN), the pathophysiology of this disorder remains unclear. Diabetes is a complex metabolic disorder and several different factors have been postulated to cause DPN. These include hyperglycemia and its metabolic consequences, oxidative stress, hypoxia due to accelerated vasculopathy, and alterations in neurotrophic factors (Vinik, 1999). None of these postulates are mutually exclusive. The most distal target fields of the long peripheral nervous system (PNS) axons are generally first affected in DPN. The sheer distance separating the nerve ending from the cell body coupled with the potential causes of DPN may render PNS neurons less resistant to trauma in diabetes. The pathology of DPN likely results from the combined contribution of axonal atrophy, demyelination, and reduced axonal regeneration (Yasuda et al., 2003; Sango et al., 2017). Recent studies clearly demonstrate a reduction in regeneration rates in both type 1 and type 2 diabetic patients compared to controls (Khoshnoodi et al., 2019), suggesting that DPN may result from delayed and incomplete regeneration and accumulating damage. Animal studies have recapitulated this delayed regeneration, demonstrating an increase in PTEN, a known inhibitor of axonal regeneration, in the nerves of diabetic mice (Pham et al., 2018). This reduced regeneration is also apparent in *in vitro* cultures of sensory neurons from diabetic animals (De Gregorio and Ezquer, 2021). The underlying cause of this delayed regeneration is unknown. Decreased axonal transport has been demonstrated in experimentally diabetic animals (Hellweg et al., 1994; Delcroix et al., 1997; Fernyhough et al., 1998). Here, we tested whether the failure to properly transport mRNAs into peripheral nerve axons following injury contributes to defective axonal regeneration. We show that following sciatic nerve injury, dorsal root ganglion (DRG) neurons fail to upregulate the levels of ZBP1, resulting in attenuation of axonal β-actin levels and decreased axonal regeneration.

## Materials and Methods

### Cell Culture

Dissociated DRG cultures were prepared as previously described (Twiss et al., 2000). For fluorescent microscopy, neurons were cultured at low density on poly-lysine/laminin coated glass coverslips. For isolation of axons, neurons were cultured in microfluidic devices (Millipore), pre-coated with poly-lysine/laminin. Axons were isolated and tested for purity by RT-PCR as previously described (Willis and Twiss, 2011).

### Plasmid Construct

A fusion construct for ZBP1-mCherry was used, the production of which has been described previously (Farina et al., 2003; Huttelmaier et al., 2005; Donnelly et al., 2011).

### Animal Surgeries and Procedures

Use of animals conformed to the Burke-Cornell Institutional Animal Care and Use Committees (IACUC) guidelines under approved protocols.

#### Modeling Diabetes

Diabetes was experimentally induced in rats by administering a single low dose of cytotoxic streptozotocin (STZ). In each experimental series, 150 g Sprague Dawley rats were food-deprived for 4–6 h prior to STZ injection. Animals were weighed and fresh STZ prepared at a final concentration of 50 mg/ml. A 1 ml insulin syringe containing 45 mg/kg was used to inject i.p. into each rat. Rats were supplied with 10% sucrose water as the sole water source for 48 h after STZ injection in order to protect them from the sudden hypoglycemic period that occurs after the lysis of the islet cells by STZ. Body weight and glucose levels were measured at 72 h after STZ injection and at subsequent 1-week intervals (nonfasting plasma glucose levels of >250 mg/dl is defined as diabetic hyperglycemia; STZ animal levels typically range from 300 to 550 mg/dl). For blood testing, the rats were restrained in a soft towel, leaving 3–5 cm at the tip of the tail exposed for blood collection. A lancet device was pressed against the surface of the skin near the tip of the exposed tail and blood analyzed using a LifeScan OneTouch Ultra Blood Glucose Meter.

#### Sciatic Nerve Injury

For injury-conditioning and morphological studies of regeneration, rodents were subjected to sciatic nerve crush at mid-thigh as previously described (Smith and Skene, 1997). Briefly, the sciatic nerves of anesthetized animals were exposed and the right nerve was crushed twice for 15 s each using #0 Dumont forceps; the left sciatic nerve was exposed but not otherwise manipulated. To harvest tissues for immunofluorescence (IF), deeply anesthetized rats were perfused transcardially with PBS followed by 4% paraformaldehyde. Tissues were post-fixed with 4% paraformaldehyde for 2–4 h, and then cryoprotected at 4°C overnight in 30% sucrose. The tissues were processed for cryosectioning.

#### Sensory Testing

Thermal sensation and mechanical sensation were assessed using a plantar infra-red analgesia meter and Dynamic Plantar Aesthesiometer (Ugo Basile), respectively. For mechanical allodynia measures, after rats were habituated to the apparatus, the Dynamic Plantar Aesthesiometer pushed a thin filament with increasing force against the plantar surface of a hind paw from beneath and the aesthesiometer recorded the latency for paw withdrawal. The force was increased from 0 to 5 g within 10 s (ramp 0.5 g/s) and is then held at 5 g for an additional 10 s. Baseline paw withdrawal latency time was calculated as the mean of five consecutive stimulations with a 5-min interval between repetitions. For thermal hyperalgesia measurement, each hind paw was tested three times with a 10 min interval between repetitions and averaged.

#### Functional Measures of Sensorimotor Regeneration

CatWalk XT (Noldus) automated gait analysis system, which uses a high-speed camera to record the placement of paws as rats cross a glass-floored corridor, was used to assess motor function following sciatic nerve injury. Briefly, the test is performed in a darkened room to allow illumination of the platform. A high-speed camera records the animal from below the platform and captures the gait *via* localized areas of light. Contact with the floor refracts internally reflected light, which appears as an illumination of the paw. This system assessed parameters of gait, including footprint area, paw pressure, limb swing speed, and toe spread. The accompanying Catwalk XT software labels each paw and provides objective measurements of the aforementioned parameters. The experimenter is blinded to the treatment groups during gait analysis of numerical output from Catwalk XT software. Each animal is required to make three passes across the apparatus. Animals were allowed to return to their home cage before each run. Scores are reported as mean ± SEM.

### Analyses of RNAs

DRGs were placed in lysis buffer (*RNAqueous* kit, Ambion) and homogenized using a Bioruptor sonicator (Diagenode). RNA was isolated from cultured cells with a *microRNAqueous* kit (Ambion) and treated with DNase prior to analysis. One-hundred nanogram of total RNA was used as a template to generate cDNA libraries (iScript, Biorad) by reverse transcription. Gene expression was assayed by real time PCR using TaqMan FAM-labeled probes (Life Technologies) with VIC-labeled GAPDH included as endogenous controls.

### Immunostaining

Tissue sections were immersion-fixed in 4% paraformaldehyde for 2–4 h, cryoprotected overnight in 30% sucrose at 4°C, and then cryosectioned. Sections were warmed to room temperature, washed in PBS, and incubated in 20 mM glycine for 30 min followed by 0.25 M NaBH_4_ for 30 min. For cultures, coverslips were fixed in 4% paraformaldehyde for 20 min and then rinsed in PBS. IF was then performed as described (Willis et al., 2005) using chicken anti-NFH (1:1,000; Millipore) and Alexa555 goat anti-chicken (1:1,000; Invitrogen) antibodies.

### Calculation of Axonal Regeneration *In vivo*

For analyses of crushed nerves, sections were immunostained for chicken anti-NFH as above. Matched exposure images were captured proximal and distal to the crush site. Montages of each nerve were constructed to encompass ± 1.5 mm from the injury site. NF+ axons were counted in every fourth longitudinal section of the sciatic nerve using Neurolucida (MBF Biosciences) and total counts were calculated using the line transect method at −1.0, −0.5, 0, 0.5, and 1.0 mm from the center of the crush site as previously described (Donnelly et al., 2011). Some images were evaluated using a “thresholding method”. For this, montage images for axonal markers were processed for thresholding (0–50) using *ImageJ*, and the mean pixel intensity per area was then measured in 100 μm bins. The “regeneration front” was determined as the bin relative to the crush site where signal thresholds were 50% of the proximal intact nerve.

### *In situ* Hybridization

Fluorescence *in situ* hybridization (FISH)/IF to detect axonal mRNAs and proteins in cultured DRG neurons was performed with digoxigenin-labeled oligonucleotide probes as previously described (Willis et al., 2007). Epifluorescence image sets from individual experiments were matched for exposure, gain and offset. *ImageJ* was used to quantify FISH signals (pixels/μm^2^) in the cell body or the distal 200 μm of the axon. For FISH/IF of sciatic nerve sections, multiplex *in situ* hybridization was performed according to the manufacturer’s instructions (Advanced Cell Diagnostics #320850). Briefly, 16 μm-thick fresh frozen sections were fixed in 4% paraformaldehyde, treated with Proteinase K, and hybridized with β-actin specific probe (#316741). Sections were imaged by confocal microscopy (Zeiss LSM 510) and analyzed using FIJI software (National Institutes of Health) by an experimenter blind to the animal group. Four sciatic nerve sections from each animal were analyzed and averaged. Neurofilament staining was used to identify axons within the sciatic nerves and create a mask for quantification. Images were thresholded into a binary signal and the percent area of fluorescent signal was quantified.

### Quantitation of Axon Length

DRG neurons were collected 7 days following sciatic nerve crush and grown in culture for 24 h. After 24 h, cultures were fixed with 4% paraformaldehyde and then processed for fluorescence immunocytochemistry with an anti-βIII-tubulin antibody (1:500, rabbit monoclonal; Epitomics). Neurons were then imaged within each well of a 96-well plate using a flash cytometer (Trophos). Total neurite length/neuron and cell body diameter were quantified using the neurite outgrowth application in Metamorph (Molecular Devices). Representative higher magnification images were taken with an inverted epifluorescence microscope. For analyses of crushed nerves, cryosections were immunostained for neurofilament (NF) and imaged on an epifluoresence microscope. NF positive axons were counted in every fourth longitudinal section of the sciatic nerve using Neurolucida (MBF Biosciences), and total counts were calculated using the line transect method at −1.0, −0.5, 0, 0.5, and 1.0 mm from the center of the crush site (Hill et al., 2001).

### cAMP Measurement

The sciatic nerve was removed, frozen in liquid nitrogen, homogenized in ice-cold 0.1 M hydrochloric acid, and centrifuged at 13,000× *g* for 50 min at 4°C. cAMP in the supernatant was determined by a direct cAMP ELISA kit (Enzo Life Sciences). The optical density was read at 405 nm using a Microplate Reader, and the cAMP concentration was normalized to total protein.

### db-cAMP Treatment of DRG Cultures

DRG neurons cultured as above were treated with db-cAMP (2 mM), total RNA was isolated, and cDNA libraries synthesized for gene expression analysis as above. In parallel experiments, cell lysates were collected from which 20 μg of protein was separated by SDS-PAGE and analyzed by Western blot for ZBP1 protein expression (Cell Signaling #D33A2, 1:1,000).

### Statistical Analysis

All statistical analyses were performed using GraphPad Prism software version 8 (GraphPad Software Inc). Data were plotted as mean ± SEM unless otherwise indicated from at least three independent experiments from separate primary cultures. The specific statistical tests used are indicated in the figure legends.

## Results

### Adult Sensory Neurons From Diabetic Animals Grow Shorter Axons *In vitro* Following *In vivo* Injury Conditioning

**Figure 1:**
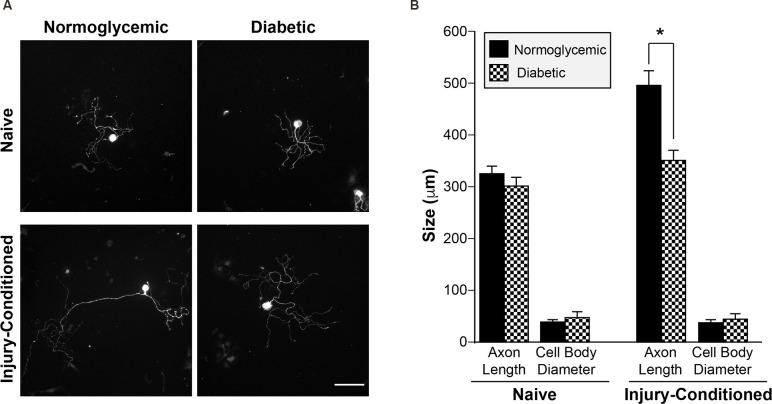
Injury-conditioned DRGs from diabetic animals have decreased regeneration. **(A)** Representative micrographs of βIII tubulin-labeled DRG neurons. 7 days after unilateral sciatic nerve crush injury, L4/L5 DRGs were removed and placed into culture. DRG neurons from diabetic animals showed attenuated axonal growth *in vitro* following conditioning-injury compared to normoglycemic controls. DRGs cultured from the contralateral, uninjured (*i.e*., naive) side showed no difference in growth. Scale bar = 100 μm. **(B)** Quantitation of mean neurite length/cell and mean cell body diameter for injury-conditioned and naive DRGs from normoglycemic and diabetic rats. *P < 0.01, Two-way ANOVA with Bonferroni post test, *n* = 1,008–1,321 neurons from six independent experiments. DRG, dorsal root ganglion.

In normoglycemic animals, an *in vivo* conditioning lesion to the sciatic nerve drives enhanced regeneration of the axons of subsequently cultured dorsal root ganglion (DRG) neurons *in vitro*. To determine whether sensory neurons from diabetic animals are able to generate a conditioning response enabling enhanced regeneration, we performed unilateral sciatic nerve crush injuries on control and diabetic animals. Seven days after crush, L4/L5 DRGs were removed and placed in culture to grow neurites for an additional 24 h. We refer to the processes of cultured DRG neurons as “axons” throughout the remainder of the text since MAP2 mRNA and protein are excluded from these processes and all processes from cultured DRGs are considered axonal in nature (Zheng et al., 2001; Donnelly et al., 2011). DRG neurons from diabetic and normoglycemic animals showed no differences in baseline axon growth *in vitro* in the absence of injury, whereas injury-conditioned DRGs from diabetic animals exhibited decreased axonal growth compared to normoglycemic rats (Figures 1A,B). This indicated that axonal growth *per se* was not deficient, but that the ability to mount the conditioning response that drives robust axonal regeneration was reduced. In addition, the size of the cell bodies in both naive and injury-conditioned DRGs did not differ in the normoglycemic and diabetic groups (Figures 1A,B). This suggests that the observed axon length differences are not due to changes in the overall composition of the DRG neuronal subtypes represented in our cultures and that the DRGs from diabetic animals do not exhibit gross morphological changes compared to control.

### Delayed Recovery of Motor Function Following Injury Is Correlated With Altered Sensory Perception in Diabetic Animals

**Figure 2:**
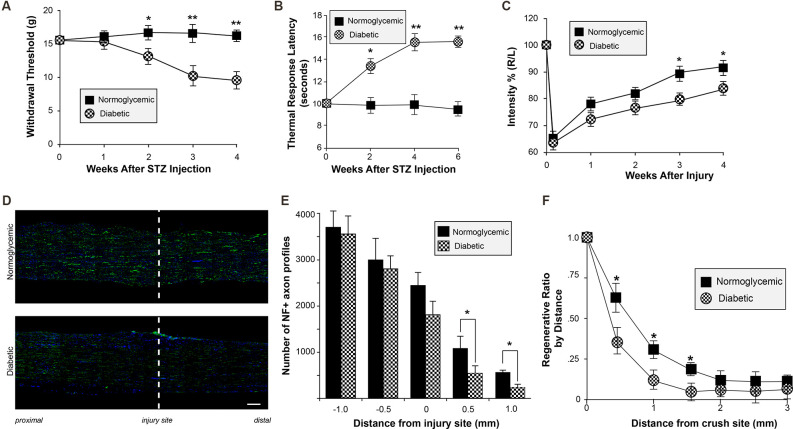
Altered sensory perception and delayed regeneration in diabetic animals. **(A,B)** Normoglycemic and STZ-injected diabetic animals were tested for altered thermal and mechanical sensory perception by plantar infra-red analgesia meter **(A)** and Dynamic Plantar Aesthesiometer **(B)** once weekly at timepoints following STZ injection. Animals developed decreased mechanical withdrawal thresholds and increased thermal response latency beginning at approximately 2 weeks after STZ injection. Animals were randomized into the control or STZ-injection group based on pretreatment withdrawal latencies to control for individual animal variation. Data are presented as mean ± SEM for 12 animals per group. *P < 0.05, **P < 0.01, Student’s t-test with Bonferroni correction after two-way repeated measures ANOVA. **(C)** Normoglycemic and STZ-injected diabetic animals were tested for functional recovery following sciatic nerve crush injury using CatWalk analysis once weekly following sciatic nerve crush. Hind paw pressure, comprising the mean intensity of the contact are on the hind paw at the moment of maximal paw-floor contact, was measured for both the injured (Right; R) and uninjured (Left; L) paws. Data are expressed in arbitrary units as the ratio between R/L, and are normalized to pre-injured levels (expressed as 100%). *P < 0.05, Student’s t-test with Bonferroni correction after two-way repeated measures ANOVA. **(D)** Representative exposure matched montage images of longitudinal sections of crushed sciatic nerves from normoglycemic and diabetic rats are shown for neurofilament (NF; green) immunoreactivity and DAPI (blue) staining; injury site is indicated by the dashed line (scale bar = 200 μm). **(E)** The number of NF-positive axons as in **(D)** was evaluated at 7 days post-nerve crush at 0, 0.5, and 1.0 mm proximal and distal to the injury site. The graph represents the mean ± SEM of the total number of NF-positive axons at each distance. **(F)** A regeneration index can be calculated by determining the distance at which the percent area of axonal staining reaches 50% compared to staining at the crush site. The distance of the regenerating front is shown by calculating the regeneration ratio for the percent of the area with axonal staining at each distance compared to the crush site.*P < 0.05, Student’s t-test, *n* = 8/group. STZ, streptozotocin.

Mechanical allodynia and thermal hyperalgesia are common in the human diabetic population and in the STZ animal model. To confirm that our diabetic rats developed altered sensation, mechanical sensation thresholds were measured after induction of diabetes. Consistent with previous studies characterizing diabetic neuropathy, our animals developed decreased mechanical withdrawal thresholds consistent with diabetic neuropathy at approximately 2 weeks after STZ injection (Figure 2A). Similar changes in thermal sensation were also observed (Figure 2B). To determine if altered peripheral sensitivity was accompanied by changes in the ability of the neurons to regenerate after injury, unilateral sciatic nerve crush injury was performed. CatWalk behavioral analysis revealed a delayed recovery of function following injury, with continued deficits in paw placement intensity up to 4 weeks following injury (Figure 2C). This delayed recovery of motor behavior correlated with decreased axonal regeneration *in vivo* following sciatic nerve injury. Histological examination of sciatic nerves following crush injury further demonstrates the reduction in the number of regenerating axons in diabetic animals compared to normoglycemic controls (Figures 2D–F). In diabetic rats, there were significantly fewer axons at points distal from the crush site compared to control animals. Taken together, these data support the conclusion that axon regeneration is decreased following injury in diabetic rats. This is consistent with a recent study from the Zigmond lab showing regenerative deficits in diabetic STZ mice (Niemi et al., 2017). It is important to note that the decrease in *in vivo* regeneration may also be consistent with the effect of the diabetic condition on Schwann cell redifferentiation and myelination.

### Reduced Levels of Axonal β-Actin mRNA in DRG Neurons From Diabetic Animals

**Figure 3:**
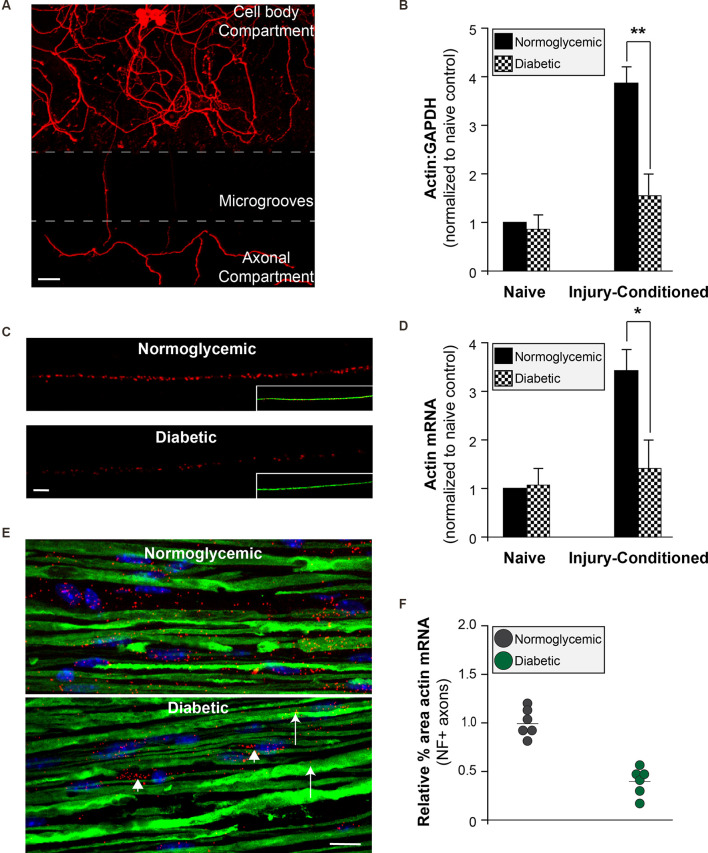
Reduced axonal β-actin mRNA levels in diabetic animals. **(A)** Adult DRGs from normoglycemic or diabetic animals were cultured in microfluidic devices to isolate pure axonal RNA (scale bar = 50 μm). **(B)** Axonal mRNA levels for β-actin from injury-conditioned or naive DRGs from normoglycemic or diabetic animals. There is a significant decrease in the axonal levels of β-actin from diabetic animal only following injury-conditioning. **P < 0.001, Two-way ANOVA with Bonferroni post-test (*n* = 6/group). **(C)** Representative micrographs of FISH for β-actin mRNA (red) following injury-conditioning in normoglycemic and diabetic animals. Inset shows NF staining (green; scale bar = 10 μm). **(D)** Quantitative FISH signal intensity for axonal β-actin mRNA from DRG cultures is shown as average pixels/μm^2^ ± SEM. Axonal β-actin mRNA was significantly decreased in the diabetic cultures compared to normoglycemic following injury-conditioning. *P < 0.05, Student’s t-test, *n* = 8/group. **(E)** FISH on sciatic nerves indicates that compared to normoglycemic animals, diabetic animals have minimal β-actin mRNA (Red; arrows) in axons (NF staining; green). There is still abundant staining for β-actin mRNA in Schwann cells in the sciatic nerve (arrowheads; nuclear staining in blue). **(F)** The relative percentage of NF+ axons containing β-actin RNA signal. Neurofilament staining was used to identify axons within the sciatic nerves and create a mask for quantification. Images were thresholded into a binary signal and the percent area of fluorescent signal was quantified. Four sections per animal were imaged, quantified, and averaged. *P < 0.05, Student’s t-test, *n* = 6/group. FISH, fluorescence *in situ* hybridization.

To determine if the attenuated injury-conditioned regeneration is the result of a failure to properly localize mRNAs into the axon for subsequent translation, we cultured DRG neurons in microfluidic devices in order to isolate axonal mRNAs (Figure 3A). In purity-verified axonal cultures from injury-conditioned diabetic DRGs, there were significantly lower axonal levels of β-actin mRNA (Figure 3B). In contrast, the levels of β-actin mRNA in axons from naive DRG neurons showed no difference between the diabetic and normoglycemic animals (Figure 3B). We confirmed this with quantitative fluorescent *in situ* hybridization (FISH) of DRGs cultures (Figures 3C,D) and sciatic nerves (Figures 3E,F) which also showed a significant reduction of axonal β-actin mRNA signal in diabetic animals compared to normoglycemic controls following injury-conditioning. Likewise, β-actin mRNA signal was not significantly different in naïve axons, indicating that the diabetic animals failed to upregulate axonal β-actin mRNA in response to injury (Figure 3D). Decreased axonal levels of β-actin mRNA following injury in diabetic animals may be indicative of a global decrease in β-actin mRNA levels. We tested for this by performing quantitative FISH of DRG soma and found similar β-actin mRNA signals in each group following injury (not shown). This was further confirmed by real time PCR measurement of β-actin expression in RNA isolated from DRG cultures (not shown).

### Levels of the β-Actin mRNA RNA-Binding Protein ZBP1 Are Attenuated in Diabetic Animals Following Injury

**Figure 4:**
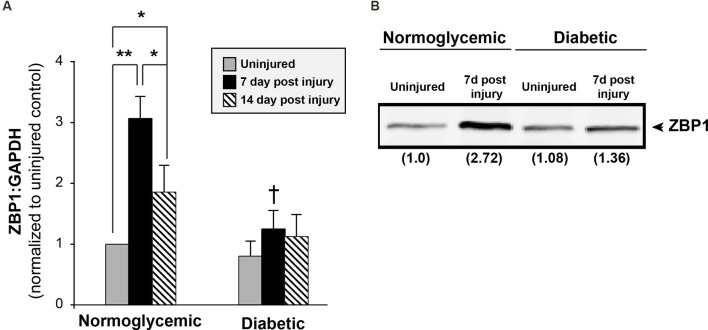
Injury does not increase ZBP1 levels in diabetic animals. **(A)** ZBP1 mRNA levels in DRGs from normoglycemic or diabetic animals following sciatic nerve crush injury. In normoglycemic animals, injury results in a transcriptional increase in the levels of ZBP1. DRGs from diabetic animals do not exhibit this post-injury increase. **P < 0.001, *P < 0.05, Two-way ANOVA with Bonferroni post test (*n* = 6/group); ^†^*P* < 0.001 vs. 7 day normoglycemic. **(B)** Western blot analysis from DRG lysates from normoglycemic or diabetic animals following sciatic nerve crush injury indicates a nearly three-fold increase in ZBP1 protein levels at 7 days following injury in normoglycemic animals. Fold induction vs. control is indicated in parenthesis.

That the abundance of axonal level of β-actin mRNA levels was reduced following injury in diabetic animals, but total levels remained unchanged, suggested that axonal localization of β-actin was attenuated. β-actin mRNA is transported through the activity of its RNA-binding protein ZBP1. To determine if the levels of ZBP1 are altered in diabetic animals, we measured the ZBP1 mRNA levels in DRGs from normoglycemic and diabetic animals at various times following sciatic nerve crush injury (Figure 4A). In normoglycemic animals, ZBP1 levels are low in uninjured DRGs and are increased approximately 3-fold by 7 days following an injury. By 14 days after an injury, the ZBP upregulation is reduced to a two-fold increase over uninjured. In diabetic animals, while the uninjured levels of ZBP1 are not significantly lower than the levels in uninjured DRGs from normoglycemic animals, injury-conditioning failed to upregulate ZBP1. We see a similar response in the ZBP1 protein levels (Figure 4B). This suggests that reduced ZBP1 following injury may limit the ability to transport cargo RNAs, such as β-actin mRNA, reducing regeneration in diabetic animals.

### Limited Levels of ZBP1 Account for Reduced Axonal Levels of β-Actin mRNA and Decreased Axonal Growth in Diabetic DRGs

**Figure 5:**
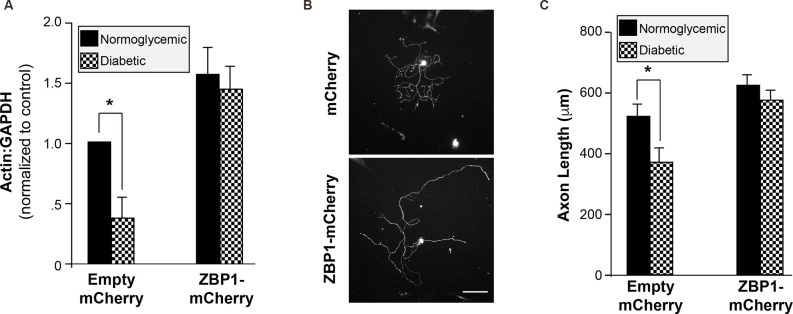
Increased availability of ZBP1 rescues axonal β-actin mRNA levels and axonal growth. **(A)** Axonal mRNA levels for β-actin from injury-conditioned DRGs from normoglycemic or diabetic animals transfected with a ZBP1-mCherry fusion construct or mCherry control construct. Overexpression of ZBP1 results in increased axonal levels of β-actin mRNA in DRGs from normoglycemic and diabetic animals. There is no significant difference in the axonal levels of β-actin mRNA in DRGs from diabetic animals when ZBP1 is overexpressed. **(B)** Representative micrographs of βIII tubulin-labeled DRG neurons transfected with the control construct (mCherry) or the ZBP1 construct (ZBP1-mCherry). Scale bar = 100 μm. **(C)** Quantitation of mean neurite length/cell for DRGs from injury-conditioned normoglycemic and diabetic as in **(B)**. *P < 0.01, Two-way ANOVA with Bonferroni post test.

Since diabetic animals fail to upregulate the levels of ZBP1 following injury and exhibit decreased regeneration following injury, we investigated whether increasing ZBP1 could rescue axonal β-actin mRNA and facilitate axonal growth in DRG neurons cultured from diabetic animals. We transfected primary injury-conditioned DRG cultures with a ZBP1-mCherry fusion protein construct. After 2 days *in vitro* to allow for the expression of the construct, the ZBP1 transfection completely corrected the axonal β-actin mRNA deficit in DRGs from diabetic animals (Figure 5A). Similarly, the attenuation of growth in the cultures was corrected (Figures 5B,C).

### cAMP-Induced Expression of ZBP1 Is Lost in Diabetic Animals

**Figure 6:**
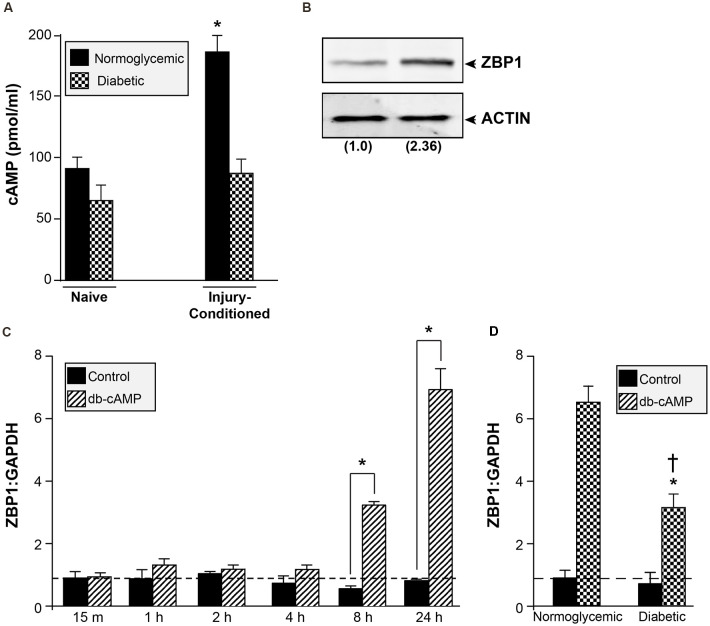
cAMP increases ZBP1 levels. **(A)** cAMP content in sciatic nerves of normoglycemic and diabetic animals. There is a significant attenuation of the cAMP increase following injury in diabetic animals. *P < 0.01 vs. diabetic injury conditioned and vs. normoglycemic naive, Student’s t-test, *n* = 6/group. **(B)** ZBP1 protein levels from DRG neurons were assayed by western blot analysis after 24 h of treatment with db-cAMP (2 mM). Fold induction vs. control is indicated in parenthesis. **(C)** Cultured primary DRG neurons from normoglycemic animals were treated with 2 mM db-cAMP for the indicated time and assessed for ZBP1 mRNA levels. There is a significant increase in ZBP1 levels at 8 and 24 h after treatment. *P < 0.01, Two-way ANOVA with Bonferroni post test, *n* = 6/group. **(D)** Cultured primary DRG neurons from normoglycemic or diabetic animals were treated with 2 mM db-cAMP for 24 h and assessed for ZBP1 mRNA levels. The levels of ZBP1 are significantly increased in neurons from diabetic animals treated with db-cAMP compared to untreated; however, the levels are significantly lower compared to neurons from normoglycemic animals treated with db-cAMP. *P < 0.01 vs. diabetic untreated, ^†^*P* < 0.01 vs. normoglycemic plus db-cAMP, Two-way ANOVA with Bonferroni post test, *n* = 6/group.

Peripheral axon injury increases cAMP levels and activates the transcription of regeneration-associated genes; a response that is required for the regeneration of sensory neurons (Ma and Willis, 2015). cAMP levels in sciatic nerves of diabetic rats are significantly lower than normoglycemic animals (Shindo et al., 1993a, b). To determine if diabetic animals failed to increase cAMP levels following injury, we measured cAMP in sciatic nerves of normoglycemic and diabetic rats before and after injury. Consistent with previous reports, diabetic animals showed a trend toward reduced basal levels of cAMP, though this did not reach significance (Figure 6A). Following injury, however, there was a significant reduction in the injury-induced cAMP upregulation in sciatic nerves from diabetic animals compared with control normoglycemic animals (Figure 6A). We next tested whether ZBP1 levels could be altered in response to cAMP. DRGs treated with db-cAMP for 24 h showed increased levels of ZBP1 protein compared to untreated DRGs (Figure 6B). Consistent with this, we recorded an increase in ZBP1 mRNA levels after 8 h of db-cAMP treatment (Figure 6C). To test whether the failure to upregulate ZBP1 in DRGs from diabetic animals is due to the lack of elevated cAMP levels, we treated DRGs from diabetic animals with db-cAMP. After 24 h of db-cAMP treatment, there was a significant increase in ZBP1 mRNA levels compared to untreated (Figure 6D). Interestingly, ZBP1 levels did not reach those of DRGs from normoglycemic animals treated with db-cAMP for 24 h.

## Discussion

Despite the high incidence, morbidity and mortality, and economic burden of diabetic peripheral neuropathy (DPN), the pathophysiology of this disorder remains unclear. Several candidates have been postulated to cause DPN, including hyperglycemia and its metabolic consequences, oxidative stress, hypoxia due to accelerated vasculopathy, and alterations in neurotrophic factors (Vinik, 1999). Decreased axonal transport has also been reported in animal models of diabetes (Hellweg et al., 1994; Delcroix et al., 1997; Fernyhough et al., 1998), and could contribute to DPN pathophysiology. The most distal targets of the smallest PNS axons (e.g., nociceptive fibers) are generally first affected in DPN (Anand et al., 1994); the sheer distance separating the nerve ending from the cell body, coupled with the potential causes of DPN, may render PNS neurons less resistant to trauma in diabetes. Here, we demonstrate that axonal mRNA transport into these distal axons is compromised in diabetes, providing evidence in support of a previously unreported mechanism that could reduce the health of peripheral neuron axons.

In normoglycemic animals, an *in vivo* conditioning lesion to the sciatic nerve facilitates the subsequent regeneration of DRG axons. Here we show that diabetic animals appear unable to mount the full conditioning response that enables enhanced regeneration. Although injury-conditioned DRGs showed decreased axonal growth, uninjured DRG neurons showed no difference in growth, providing evidence that the ability to generate a conditioning response is reduced by diabetes. Since DRG cell-body sizes in both naive and injury-conditioned rats did not differ in the normoglycemic and diabetic groups, this suggests that axonal abnormalities are not due to changes in the overall composition of the DRG neuronal subtypes represented in our cultures; however, a more detailed analysis of the neuronal subtypes is required in order to completely discount this as a possibility. A regenerative deficit is also evident *in vivo* following injury, as both functional and morphological measurements of regeneration are attenuated following injury in diabetic animals. We have previously shown that regenerating axons of injury-conditioned DRG neurons are capable of local protein synthesis during rapid axonal regeneration and that this axonal protein synthesis is altered in response to injury signals (Willis et al., 2005, 2007). Thus, the failure to properly mobilize mRNAs into the axons in response to injury has the potential to lead to deficits in axonal regeneration (Donnelly et al., 2011; Willis et al., 2011).

We have previously shown that ZBP1 levels are a limiting factor for regeneration. The present study, however, is the first report of a failure to upregulate ZBP1, leading to reduced axonal regeneration in a pathophysiological condition. The induction of gene expression by axon injury provides evidence for an active process by which injured neurons sense axonal damage to drive an adaptive response. This could be achieved by the disruption of the retrograde flow of target-derived trophic signals (i.e., loss of NGF; Raivich et al., 1991; Gold et al., 1993), activation of existing or newly synthesized local (axonal) factors that are retrogradely transported (i.e., DLK, JNK, STAT3, CREB; Hanz et al., 2003; Cavalli et al., 2005; Cox et al., 2008; Ben-Yaakov et al., 2012; Shin et al., 2012), and depolarization of the axon due to the disruption of the plasma membrane (i.e., Ca^2+^ influx leading to cAMP elevation; Ghosh-Roy et al., 2010; Cho et al., 2013). Ultimately, the arrival of these signals at the cell body would drive transcriptional changes that initiate a regenerative response. Whereas reduction in cAMP levels in the sciatic nerve of diabetic animals has been previously reported (Shindo et al., 1993a, b), here we show that this deficit is exacerbated following injury by a failure of cAMP to upregulate in response to injury. Reduced cAMP in peripheral nerves has been postulated to play a role in the pathogenesis of diabetic peripheral neuropathy, and here we present a possible mechanism for how the loss of cAMP can impact axonal function. It remains to be determined why cAMP levels in peripheral nerves are suppressed under diabetic conditions and the mechanism through which cAMP drives ZBP1 expression. In addition, when treated with db-cAMP in culture, DRGs from diabetic animals still do not upregulate the levels of ZBP1 to the same degree as those from normoglycemic animals. This suggests that the suppression of cAMP levels in the sciatic nerve of injured diabetic animals may account for some of the failure to upregulate ZBP1 levels. Additionally, it is likely that the neurons themselves are less responsive to the signal generated by cAMP. Understanding how the level of ZBP1 is regulated, and why its regulation fails following injury in the diabetic state, may provide new strategies to enhance axonal regeneration and overcome deficits that lead to peripheral neuropathy. Interestingly, there are considerable differences in many of the behavioral outcomes in diabetic rodent models, especially regarding thermal stimulation, with studies showing reduced, increased, or unchanged latencies (Miranda et al., 2021). The cause of these differences is unknown; however, variation in the experimental conditions, the strain of rodent used, or the age of the animals have all been postulated to explain these discordant results. Though the face validity of these rodent models is not necessarily recapitulated in this regard, the alteration in sensory processing is evident. It would be interesting to determine the extent to which these different perturbations in models, with their associated differences in thermal latency changes, correlate with altered dysregulation of ZBP1 levels and RNA axonal localization. In addition, RNA binding proteins other than ZBP1 could also be dysregulated, raising the possibility that multiple axonal RNAs are altered in the diabetic state. Though we have focused on β-actin RNA as the canonical cargo for ZBP1 in this work, we have evidence that other ZBP1-cargos, such as GAP43 RNA, are also reduced (not shown), supporting our previous demonstration that ZBP1 levels are limiting for axonal transport of a “cassette” of RNAs (Donnelly et al., 2011). RNA profiling in axons from diabetic animals would help to determine the extent to which this is a global phenomenon resulting in widespread alteration in the axonal translatome.

Absent from the results shown here is an understanding of the contribution that the Schwann cells play in both the failure to mount a full conditioning response and the subsequent reduced regeneration. These PNS glial cells are critical for the proper function of the DRG neurons, and disease states such as diabetes have been shown to alter their function, resulting in neurodegeneration and pain. Indeed, evidence of mild axonal demyelination and remyelination is apparent even when the axon is normal, suggesting that Schwann cell abnormalities may be an early step in DPN pathology, and might be a primary cause of axonal damage (Malik et al., 2005; Gonçalves et al., 2018; Placheta-Györi et al., 2021). Experimental evidence supports a primary role for dysfunctional Schwann cells in the etiology of DPN. Exosomes isolated from Schwann cells grown in high glucose were sufficient to cause reduced axonal growth *in vitro* and the appearance of peripheral neuropathy characteristics *in vivo* (Jia et al., 2018). Given this, Schwann cell-derived exosomes are an emerging interest as potential therapeutic approaches to treating DPN. A recent study by Poplawski and colleagues shows that Schwann cells can act to regulate gene expression in DRGs, especially those considered part of the RAG response and that this gene expression is altered when the Schwann cells are abnormal (Poplawski et al., 2018). These results point to a fundamental role for Schwann cell-sensory neuron communication in driving the regeneration response and may account for the failure to mount the conditioning response and upregulate ZBP1 shown here.

The prevailing theories for the underlying mechanism(s) driving diabetic peripheral neuropathy (DPN) are that either a dying back degeneration of axons or the accumulation of injuries along the length of the axon causes the distal-to-proximal progression that is common in DPN. In the first mechanism, the primary insult is thought to consist of impairment of synthesis and axonal transport of proteins, both in the anterograde and retrograde directions (Hellweg and Hartung, 1990; Delcroix et al., 1997; Fernyhough et al., 1998). This would naturally impact the longest axons of the body, and the most distal aspects of those axons, which are necessarily most dependent on proper axonal transport. The alternative possibility for the proximal-to-distal presentation is the accumulation of multiple injuries along the length of the axon (Dyck and Giannini, 1996; Thomas, 1999). Again, the longest axons would be more likely to accumulate these types of local injuries, resulting in their early dysfunction. The first mechanism assumes that it is loss of axonal *protein* transport that is the primary driver of axonal damage, rather than transport of alternative cargos such as mRNAs. The second mechanism does not account for the inability of the diabetic axons to repair injuries along the length of the axon, a process that under normal conditions is relatively robust. In addition, while evidence supporting either of these potential mechanisms exists, no emergent unifying theory encapsulating both of these possibilities while also accounting for the limitations described above has been described. Our data shown here account for both mechanisms as the underlying cause of DPN and postulate a novel role for mRNA axonal transport in DPN.

## Data Availability Statement

The raw data supporting the conclusions of this article will be made available by the authors, without undue reservation.

## Ethics Statement

The animal study was reviewed and approved by Weill Cornell Medical College Institutional Animal Care and Use Committee.

## Author Contributions

DW is the guarantor of this work and, as such, had full access to all data in the study and takes responsibility for the integrity of the data and the accuracy of the data analysis. JJ performed experiments, collected data, and wrote the manuscript (Figures 1–3). CJC performed experiments and collected data (Figure 3). CC performed experiments, collected data, and reviewed/edited the manuscript (Figures 1–4). DG, MP, and MC performed experiments and collected data (Figure 2). WM performed experiments, contributed to discussion and planning of experiments (Figure 5). TM performed experiments, collected and analyzed data, contributed to discussion and planning of experiments, and reviewed/edited the manuscript (Figures 1, 6). DW researched data and wrote the manuscript (Figure 6). All authors contributed to the article and approved the submitted version.

## Funding

This work was supported by grants from the American Diabetes Association (1-12-BS-227; DW), NIH (National Institute of Nursing Research; NR010797; DW), and the Burke Foundation (DW).

## Conflict of Interest

The authors declare that the research was conducted in the absence of any commercial or financial relationships that could be construed as a potential conflict of interest.

## Publisher’s Note

All claims expressed in this article are solely those of the authors and do not necessarily represent those of their affiliated organizations, or those of the publisher, the editors and the reviewers. Any product that may be evaluated in this article, or claim that may be made by its manufacturer, is not guaranteed or endorsed by the publisher.
